# Primary Ovarian Mesothelioma: A Case Series with Electron Microscopy Examination and Review of the Literature

**DOI:** 10.3390/cancers13092278

**Published:** 2021-05-10

**Authors:** Luigi Vimercati, Domenica Cavone, Maria Celeste Delfino, Biagio Bruni, Luigi De Maria, Antonio Caputi, Stefania Sponselli, Roberta Rossi, Leonardo Resta, Francesco Fortarezza, Federica Pezzuto, Gabriella Serio

**Affiliations:** 1 Interdisciplinary Department of Medicine, Occupational Medicine-Section Ramazzini, University of Bari Aldo Moro, 70124 Bari, Italy; luigi.vimercati@uniba.it (L.V.); domenica.cavone@uniba.it (D.C.); maria.delfino@uniba.it (M.C.D.); luigi.demaria@uniba.it (L.D.M.); antonio.caputi@uniba.it (A.C.); stefania.sponselli@uniba.it (S.S.); 2 Ultrastructure Laboratory, Istituto Superiore di Sanità, 00161 Rome, Italy; biagio.bruni@iss.it; 3 Department of Emergency and Organ Transplantation (DETO), Pathological Anatomy Section, University of Bari Aldo Moro, 70124 Bari, Italy; roberta.rossi@policlinico.ba.it (R.R.); leonardo.resta@uniba.it (L.R.); 4 Department of Cardiac, Thoracic, Vascular Sciences and Public Health, University of Padova Medical School, 35121 Padova, Italy; francesco.fortarezza@aopd.veneto.it

**Keywords:** mesothelioma, ovarian mesothelioma, asbestos, talc, misdiagnosis

## Abstract

**Simple Summary:**

Primary ovarian mesothelioma is a rare and aggressive neoplastic disease with a poor prognosis. The rarity of this entity and the challenging differential diagnosis with other ovarian and peritoneal neoplasms may lead to frequent misdiagnosis and some concerns about its histogenesis. This case series describes four histologically and ultrastructurally documented primary ovarian mesotheliomas in exposed patients, as reported in the medical history. Because of the few cases described, we reviewed the English literature on ovarian mesothelioma and on its possible risk factors, already known and recognized for pleural, pericardial, peritoneal, tunica vaginalis mesothelioma. Describing such rare cases and summarizing the knowledge so far is fundamental to gain greater awareness of this neoplasm and try to answer unsolved questions on its origin.

**Abstract:**

Primary ovarian mesothelioma is a rare, aggressive neoplastic disease with a poor prognosis. At onset, the tumor is only rarely limited to the ovaries and usually already widespread in the peritoneum. The rarity of this entity and the difficulties differentiating it from either ovarian carcinoma or peritoneal mesothelioma may lead to frequent misdiagnoses and may raise some concerns about its histogenesis. Thus, reporting such rare cases is fundamental to gain greater awareness of this neoplasm and try to answer unsolved questions. Herein, we described four cases of histological diagnoses of ovarian mesothelioma extrapolated by the regional mesothelioma register of Apulia (southern Italy). In all cases, a detailed medical history was collected according to national mesothelioma register guidelines. A broad panel of antibodies was used for immunohistochemistry to confirm the diagnoses. Moreover, ovarian tissue samples were also examined by transmission and scanning electron microscopy, detecting asbestos fibers and talc crystals in two cases. Because of the few cases described, we reviewed the English literature in the Medline database, focusing on articles about ovarian mesothelioma “misclassification”, “misdiagnosis”, “diagnostic challenge” or “diagnostic pitfall” and on unsolved questions about its histogenesis and possible risk factors.

## 1. Introduction

Mesothelioma is a rare malignancy that usually arises in the pleura and less commonly in the peritoneum or rare sites, such as the pericardium and gonads [1,2,3]. From the data of the Italian National Mesothelioma Registry (ReNaM) in the period from 1993 to 2015, 79 out of 27,356 cases of mesotheliomas (0.3%) affected the vaginal tunic (VI report ReNaM) [4]. In women, a primary gonadal mesothelioma can originate from the germinative epithelium that is located in the most superficial portion of the organ and represents a specialization of peritoneum that, passing from the mesovary on the surface of the ovary, turns into an epithelium of the cubic type [5].

Due to its anatomical constitution, the female peritoneum is vulnerable to the entry of carcinogenic agents through the reproductive organs [6,7,8]. Although the etiology of cancer malignancies is supposed to be multifactorial [9], asbestos fibers and contaminated talc can contribute to neoplastic degeneration [10,11,12,13].

As mesothelioma and other ovarian epithelial neoplasms share the same cell of origin, the differential diagnosis between epithelial ovarian cancers and mesotheliomas is difficult with overlapping radiological findings and morphological features [14]. Pathogenetic mechanisms for serous ovary carcinoma are well defined. High-grade forms arise from tubal-type epithelium that can be found in the fallopian fimbria and ovarian surface or inclusion cysts lined by ovary epithelium [15]. Instead, for ovarian mesothelioma, the pathogenetic pathways remains unclear. 

Since the end of the late 1990s, immunohistochemistry has made a strong contribution to identifying the correct neoplastic phenotype with the introduction of sensible and specific antibodies [16,17]. Moreover, even though electron microscopy (transmission electron microscopy—TEM) is an ancillary technique mainly used in the field of scientific research [18], it may continue to have a purpose in the pathological diagnosis of difficult cases, as also reported in the latest statement for the pathological diagnosis of mesothelioma [19]. In fact, with ultrastructural examination, it is possible to identify cellular details that reveal the mesothelial origin, such as elongated and branching microvilli as well as giant desmosomes and evident intracytoplasmic tonofilaments [20,21,22,23,24]. Scanning electron microscopy (SEM) equipped with an X-ray microanalysis system (EDS) on pathological specimens may also be considered as an additional tool for the research of fibers or crystals eventually attributable to a previous exposure history.

Awareness of the existence of this rare form of mesothelioma is important to prevent misdiagnosis and for consequences in terms of therapeutic treatment [25,26,27].

Herein, we described four cases of ovarian mesothelioma reported in the regional mesothelioma register of Apulia (southern Italy) and histologically documented. A broad panel of antibodies was used for immunohistochemistry to confirm the diagnoses. Ovarian tissue samples were also examined by transmission and scanning electron microscopy to deepen both the diagnosis and the possible previous exposure to known risk factors for mesothelioma of other sites.

Given the rarity of the tumor and the numerous debates on the topic, we reviewed the English literature in the Medline database, focusing on articles about ovarian mesothelioma “misclassification”, “misdiagnosis”, “diagnostic challenge” or “diagnostic pitfall” and on unsolved questions.

## 2. Materials and Methods

### 2.1. Medical Records and Questionnaires for the Assessment of Exposure to Asbestos

From the review of the Apulia Mesothelioma Register through the ReNaM from 1995 to 2017, four cases had been diagnosed as ovary mesotheliomas [4]. An informed consent for revision and further analyses was collected before the inclusion in the study. Patients’ medical records were carefully reviewed to assess exposure to asbestos according to the guidelines of the National Mesothelioma Register [28]. Work, family, residential history, habits of life, habits of free time, any domestic exposures and personal hygiene attitudes were thoroughly investigated.

### 2.2. Histological Findings and Immunohistochemical Staining

In all 4 cases initially diagnosed with primary ovarian mesothelioma, hematoxylin and eosin histological slides as well as formalin-fixed and paraffin-embedded tissue samples obtained at the time of diagnosis were available for re-evaluation. According to diagnostic guidelines [19,29], a broad panel of antibodies was used for immunohistochemistry analysis using calretinin (Pab, Zymed, San Francisco, CA, USA), BAP1 (BRCA1-associated protein 1, clone C4, Santa Cruz, 1:50), CEA (Carcino-Embryonic Antigen, Pab, preluited, NeoMarker, Fremont, CA, USA), MOC 31 (Epithelial Specific Antigen/Ep-CAM, (Mab, preluited, DAKO, CA, USA), WT1 (Wilm’ Tumor-1, clone WT49, Novocastra, dilution 1:40) and Podoplanin (clone D2-40, preluited, DAKO). Immunohistochemical analyses were carried out using the Envision Detection System (Dako, Glostrup, Denmark) with diaminobenzidine (DAB) as chromogenic substrate, on the Dako Techmate automatic stainer (Carpinteria, CA, USA). For fluorescence in situ hybridization (FISH) analysis, the FISH locus specific CDKN2A (9p21) probe (Abbott, Abbott Park, IL, USA) was used, as previously described [30].

### 2.3. Transmission and Scanning Electron Microscopy

Ultrastructural examination was performed as previously described [18]. Briefly, after fixation in 2.5% glutaraldehyde and an overnight wash in the same buffer, the samples were post-fixed with 1% osmium tetroxide in PBS for 2 h at 4 °C, before being processed for embedding in Epoxy Resin-Araldite(M) CY212 (TAAB, Aldermaston, UK). Semi-thin sections 2 μm thick were stained with Toluidine blue. Images of semi-thin sections were captured using a Nikon photomicroscope equipped with a Nikon Digital sight DS-U1 camera (Nikon Instruments SpA, Calenzano, Italy). Ultra-thin sections were mounted on Formvar-coated nickel grids and stained routinely with uranyl acetate and lead citrate. Ultra-thin sections were observed using a transmission electron microscope Morgagni 268 (FEI Company, Milan, Italy).

The search for fibers in ovarian paraffin-embedded tissues was performed with the aid of scanning electron microscopy (SEM) (SEM XL 30 of the FEI) [31,32,33,34]. The extraction methodology was performed as opportune [35,36,37]. Specifically, the samples analyzed appeared to be made up of small fragments of ovarian neoplastic tissue that was formalin-fixed paraffin-embedded. The samples were carefully dewaxed and dried. To study and analyze the inorganic particles within the tissues, the organic part was destroyed according to standard procedures for the digestion of the tissue, and ovarian tissue underwent a chemical attack through sodium hypochlorite digestion. Subsequently the solution was filtered on a polycarbonate membrane with a diameter of 25 mm and porosity of 0.4 µm nuclepore filters (PolyCarbonate filter). The membrane was then washed by filtering double-distilled water preheated to 60 °C to eliminate the sodium hypochlorite crystals that formed during digestion. At this point the membrane, once dried, was mounted on a suitable support, coated with an Au film and was examined by electron microscopy equipped with an energy-dispersive spectrometer.

## 3. Results

From 1995 to 2017, 138 cases of peritoneal mesothelioma were uncounted in female patients in the Apulia mesothelioma register. Four cases were classified as primary ovarian mesotheliomas based on exclusive localization on the ovary without any peritoneal spreading (stage T1b, based on TNM system of ovarian cancer).

The mean age of the patients was 52.2 years, and the mean survival was 52 months. Regarding the assessment of exposure to asbestos, the information from the Mesothelioma Register reports an average duration of exposure of approximately 24 years and an average latency of 40 years.

**Figure 1:**
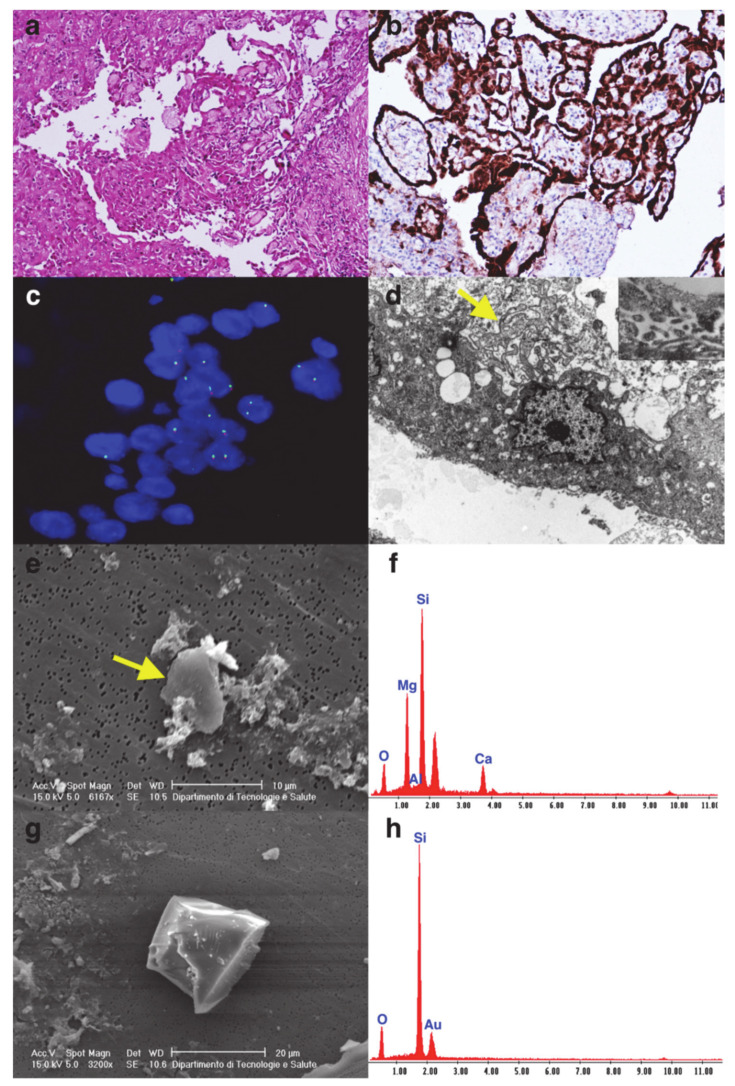
Case 1. Histological section of papillary ovarian mesothelioma ((**a**) hematoxylin and eosin stain, original magnification ×200). The neoplastic cells were strongly positive for calretinin ((**b**) immunohistochemistry, original magnification ×200). FISH analysis showed CDKN2a (p16) heterozygous deletion (**c**). Upon ultrastructural examination, the neoplastic cells showed microvilli-like expansion (arrow, inset) on the luminal surface ((**d**) original magnification ×4400; inset original magnification ×30,000). The SEM micrograph of foliated talc crystals ((**e**) arrow) and EDX analysis spectrum of the particle (**f**) were compatible with its general chemical formula (Mg_3_Si_4_O_10_(OH)_2_). SEM image of quartz crystal and EDX analysis spectrum (**g**,**h**).

**Figure 2:**
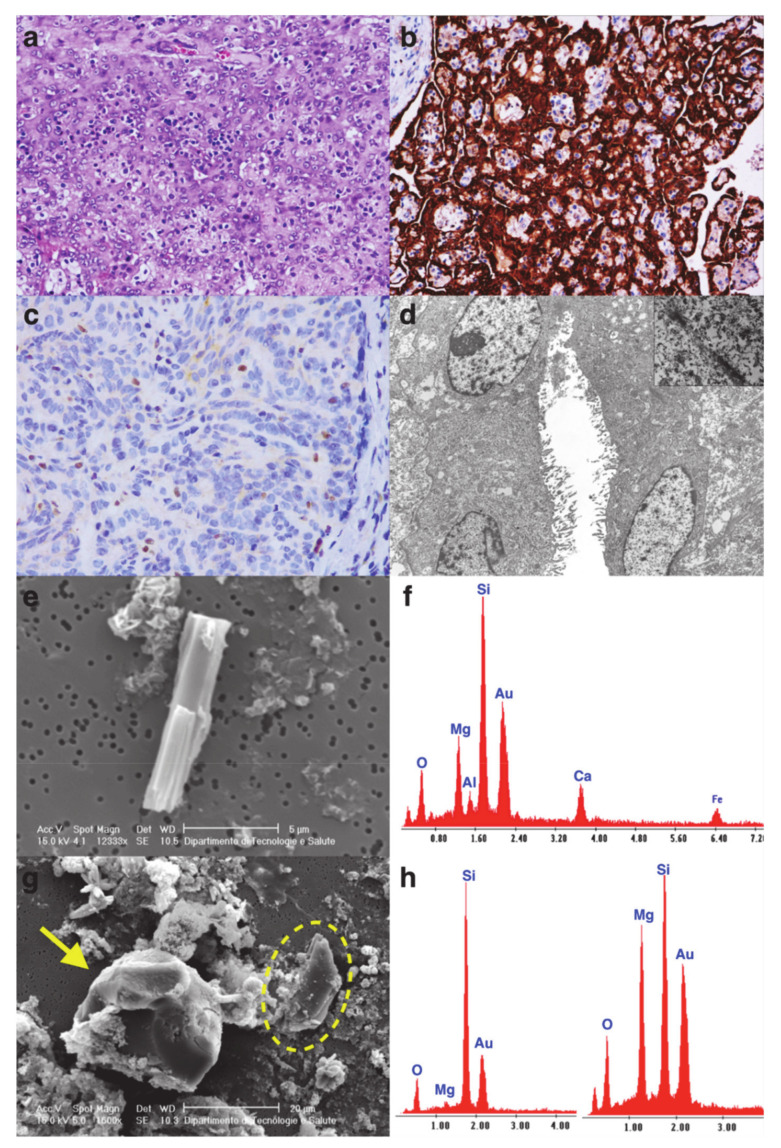
Case 2. Histological section of ovarian mesothelioma with solid and trabecular patterns ((**a**) hematoxylin and eosin stain, original magnification ×200) and diffusely immunoreactive calretinin ((**b**) immunohistochemistry, original magnification ×200). Lack of nuclear expression of BAP1 ((**c**) immunohistochemistry, original magnification ×400). The neoplastic cells showed numerous elongated and branching microvilli on the luminal surface ((**d**) original magnification ×4400). At higher magnification, desmosome-like junctions and intracytoplasmic tonofilaments were evident (inset, original magnification ×10,000). The SEM micrograph (**e**) and EDX analysis (**f**) spectrum were morphologically and chemically (chemical formula: Ca2Mg5Si8O22(OH)2) compatible with tremolite fiber. In the same specimen, quartz ((**g**) arrow) and talc crystals ((**g**) dashed lines) were also evident with the respective EDX analysis spectrum ((**h left**) quartz; (**h right**) talc crystal).

**Figure 3:**
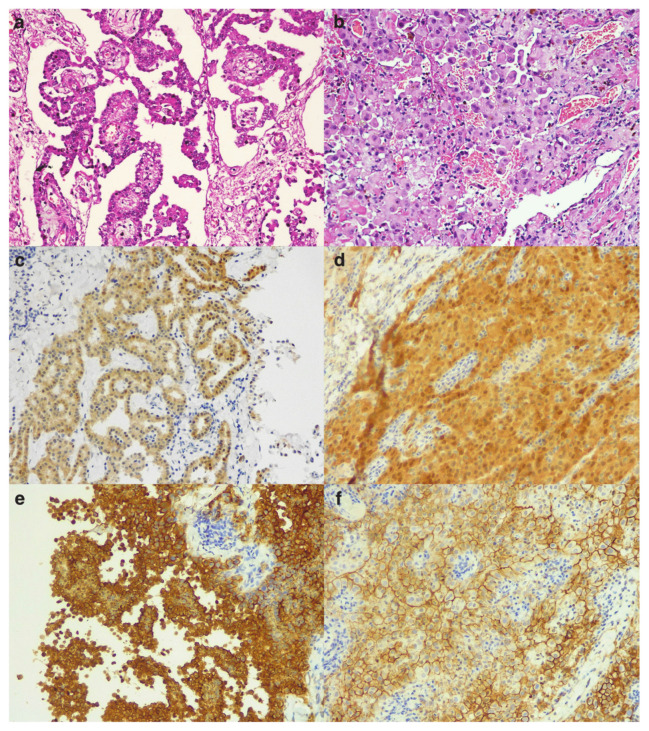
Case 3: Histological section of ovarian mesothelioma with papillary pattern ((**a**) hematoxylin and eosin stain, original magnification ×200) and diffusely immunoreactive calretinin ((**c**) immunohistochemistry, original magnification ×200), and D2-40 ((**e**) immunohistochemistry, original magnification ×200). Case 4: Histological section of ovarian mesothelioma with solid pattern and deciduoid cytology ((**b**) hematoxylin and eosin stain, original magnification ×200) and diffusely immunoreactive calretinin ((**d**) immunohistochemistry, original magnification ×200), and D2-40 ((**f**) immunohistochemistry, original magnification ×200).

The detailed data of each patient are shown in Table 1 and Table 2. In Figure 1, Figure 2 and Figure 3 some explicative images of each case are reported.

## 4. Discussion

In this study, we reported four cases of primary ovarian mesothelioma, two of which involved both ovaries [29]. Each case was histologically diagnosed, reviewed and confirmed by the immunohistochemical phenotype, with a TNM classification (T1b) which lays for primary tumors [38,39]. The mean age of the patients and the mean survival agreed with the literature [14]. Regarding the average duration of exposure and the latency, as well as known in the literature for pleural or peritoneal malignant mesothelioma, the effects are observed after long latencies with cumulative dose effects also relative to low-intensity exposures [40,41,42,43].

No tissue samples were available for electron microscope examinations in two of these cases (case 3 and 4), instead in the other two cases (case 1 and 2), ultrastructural examination (TEM) was also performed, thus highlighting some ultrastructural findings most in favor of the mesothelial origin. Furthermore, the analyses performed in SEM-EDX have generally highlighted the presence of both natural (such as asbestos, talc and silica fibers) and artificial (such as artificial glass fibers) inorganic material. This is an interesting finding in our case series, which is in agreement with the literature that has highlighted the possible role of asbestos and contaminated talc in the pathogenesis of ovarian mesothelioma, even when it is apparently not possible to trace a history of exposure. This may represent a bias, as most cases may remain unknown due to the long latency of the disease and the consequent possibility of not remembering remote exposure events (recall bias), for example, concerning the use of contaminated talc in pediatric age. This could be overcome by a careful reconstruction of exposure to asbestos, as happened in our case series that enabled us to obtain a comprehensive exposure history through a standardized approach. In case 1, only domestic exposure due to an ironing board coated with asbestos-containing material used for approximately 10 years until 1985 was documented. The case was classified as “domestic exposure” according to ReNaM code 6 as follows: subjects not professionally exposed but who have been exposed to asbestos during domestic activities (use of asbestos furnishings) (ReNaM Guidelines) [28]. It is well known that in the period between 1950 and 1980, the risk of domestic exposure to asbestos was high in houses due to the presence of products containing asbestos, especially those used for thermal insulation, such as ironing board covers, hairdryers, ovens and stoves [44,45,46]. The analyses in SEM-EDS did not detect asbestos fibers but highlighted the presence of both natural inorganic material, such as talc and quartz, and artificial such as glass fibers. With regard to the use of cosmetic talc for perineal hygiene, the subject did not remember its use but at the same time did not exclude its use, which presumably occurred when she was an infant. In case 2, it was possible to exclude occupational exposure, and no family member was professionally exposed. The only reported exposure was also domestic. The patient reported the use of cosmetic talc for perineal hygiene from birth (1933) until 1980. It is well documented that until 1976, cosmetic talc was frequently contaminated by asbestos [31,47,48,49,50,51]. SEM-EDS analyses revealed the presence of fibers probably attributable to asbestos tremolite. They also highlighted the presence of both natural inorganic material such as talc and quartz and artificial such as glass fibers. Tremolite is usually found as an accessory mineral in talc due to its common mineral origin [35,52]. This information could help complete the picture of the subject’s possible exposure. In addition, in case 3, the only reported exposure was domestic. The patient reported the use of cosmetic talc for perineal hygiene from birth (1947) until 1985. In case 4, the patient had inhaled asbestos from washing her husband’s work clothes who was occupationally exposed to asbestos. Moreover, asbestos exposure may have also occurred through the transvaginal pathway crossed by fibers during sexual intercourse [53].

According to the current WHO definition [15], “the ovaries may rarely represent the main site of (mesothelioma) involvement, mimicking a carcinoma”. Until the definition of the lineage origin by the WHO, ovarian mesotheliomas may have been reported under a wide variety of diagnostic terms (e.g., adenomatoid tumors) [54,55,56,57,58,59,60,61,62,63]. In our review, we only focused on the 11 articles that referred to ovarian mesothelioma as such (listed in Table 3) [64,65,66,67,68,69,70,71,72].

### 4.1. The Histogenesis and the Role of Electron Microscopy

The plausible histogenesis of primary ovarian mesothelioma has been debated in the literature. Endothelial, mesothelial, mesonephric and Mullerian origins have been proposed and investigated by several authors. Since the first evidence, the most accredited theory favors mesothelial origin, according to which mesothelium, stroma and glands of all types are derived from the embryonic coelomic epithelium that undergoes metaplasia into different cell lineages [6,11,59,64,73,74,75,76,77]. More recently, the ovarian surface epithelium (OSE), peritoneum and subjacent connective tissue have been demonstrated to originate from the pleuripotential embryonic coelomic epithelium and subcoelomic mesenchyme [78,79].

During embryonic development, this epithelium overlies the gonadal area. Through several proliferation and differentiation phases, it gives rise to part of the gonadal blastema. Near the gonads, invagination originates in the Mullerian (paramesonephric) ducts, the primordia for the epithelia of the oviduct, endometrium and endocervix [80]. Compared to the pelvic peritoneum, the OSE is less differentiated, and CA125, usually expressed by extraovarian mesothelium, is absent or only occasionally expressed [81].

The ultrastructural features of ovarian carcinoma and mesothelioma are similar, and they are composed of channels different in size that are lined by a single or double layer of flattened or low cuboidal cells with a papillary growth pattern. However, some morphological characteristics can be more in favor of a mesothelioma rather than an ovarian carcinoma. For example, the presence of numerous microvilli with hierarchical branching, bundles of cytoplasmic filaments and desmosomes longer than 1 µm (i.e., giant desmosomes) are most consistent with a mesothelial derivation [74,82,83]. In 1979, Blaustein et al. [84] compared the histological and ultrastructural features of ovary and pelvic peritoneum cells. These authors reported that in cases of cystadenocarcinoma of the ovary, the ovary surface cells, and the contiguous pelvic peritoneum underwent similar changes. In a subsequent study, Foyle et al. [85] found that in 10 out of 25 papillary peritoneal mesotheliomas, the histological structure was superimposable on ovary serous papillary tumors (so-called ovary papillary mesothelioma). One year later, Warhol [86] demonstrated that mesothelioma had longer and more complex microvilli than carcinomas and a greater content of tonofilaments according to electron microscopy analysis. In particular, tonofilament content is extremely useful to discriminate between ovarian carcinomas and mesotheliomas. If the tumor has features of either tubal- or intestinal-type epithelium, cilia, abundant mucin or dense core granules, it should be considered of ovarian origin [67]. Ultrastructural examination has been proven useful in distinguishing mesotheliomas from carcinomas [20,21,22,23,24]. Currently, the differential diagnosis is based on ancillary techniques, mainly a broad panel of immunohistochemical antibodies [87,88]. However, electron microscopy remains an additional tool in cases of mesothelioma arising in unusual sites or of dubious interpretation, as confirmed in the last consensus statement for the diagnosis of pleural mesothelioma [19].

### 4.2. Differential Diagnosis: The Real Challenge

Several differential diagnoses should be taken into account when dealing with mesothelial proliferation involving the ovary. Secondary involvement by peritoneal mesothelioma, a serous papillary tumor and a borderline tumor of the ovary with peritoneal deposits represent the most difficult diagnostic challenge. Their distinction is fundamental for correct patient care, as it implies different etiological considerations, clinical courses and treatments [60,85,86,89].

The risk of underestimating primary ovarian mesothelioma also exists [90]. Barber et al. [91], according to previous evidence [76,92,93], reported, in a case series, how peritoneal metastases of ovarian tumors had been misdiagnosed as multiple primary cancers. Likewise, Young et al. [94] highlighted that some rare variants of malignant mesothelioma of the female genital tract and peritoneum with ovarian involvement may be underdiagnosed.

From an etymological point of view, many synonyms have been attributed to serous surface papillary carcinomas (SSPCs) originating from the ovaries, including “ovarian mesothelioma”. A multicentric and synchronous origin from peritoneum and ovarian surfaces has also been speculated [95]. Based on a continuous histological spectrum of peritoneal and ovarian tumors in women, ranging from epithelial malignant mesothelioma to SSPC, it is not surprising that such misclassifications and erroneous attributions of the site of origin of the tumor occur, mainly when diagnostic tools are lacking.

Several cases of secondary involvement of the ovary by diffuse malignant peritoneal mesothelioma have been reported in the literature; more rarely, ovarian primary malignant mesothelioma may involve the peritoneum [81,82]. Indeed, clinical and morphological distinction between some entities (e.g., well-differentiated peritoneal mesothelioma or florid peritoneal mesothelial proliferations) may be difficult due to some overlapping morphological and immunohistochemical (IHC) features, including papillary structures, clinical symptoms and imaging findings [96,97,98,99,100,101,102,103,104,105,106,107,108,109,110,111,112,113]. This is mainly true if we also consider the new knowledge on the emerging entity of the mesothelioma in situ that can remain hidden for a long time [114,115].

Nonetheless, some histological clues helpful for defining the tumor origin have been searched [69]. The diagnosis of malignant mesothelioma may be favored by the detection of extensive tumor necrosis, marked cytological atypia, absence of marked nuclear pleomorphism, absence of a high mitotic rate, focal biphasic growth pattern, infiltrative growth pattern, prominent tubulopapillary pattern, polygonal cells with eosinophilic cytoplasm and the presence of intracellular acid (PAS negative) mucin rather than neutral (PAS positive) mucin. Likewise, psammoma bodies, hierarchical branching of papillae, higher cellular stratification, detached cell clusters, higher nuclear atypia with frequent anaplastic or bizarre nuclei, abnormal mitotic figures and higher mitotic rates are more in favor of serous carcinoma [112,116].

To date, the gold standard for the correct diagnosis is the definition of the immunohistochemical phenotype. For mesotheliomas arising in other sites, the final diagnosis of mesothelioma must be achieved by the combination of positive and negative markers of mesothelial origin [16,19,20,21,22,26,88,97,109,111,113,117,118,119,120,121,122,123,124,125,126,127]. The International Mesothelioma Panel recommends that at least two mesothelial markers and two markers for other tumors (dependent on the differential diagnosis based on morphology) with a sensitivity and specificity greater than 80% should be performed [128]. Mesothelial cells are generally positive for calretinin, thrombomodulin, cytokeratin 5/6, WT1, D2-40, vimentin, HBME-1 and CD44H, while polyclonal and monoclonal carcinoembryonic antigen, Ber-EP4, Leu-M1, CA-125, B72.3, PAX8, MOC 31, CA19-9 and ER are the most common markers of carcinoma. Comin et al. [129] reported that H-CD h-caldesmon, calretinin, ER estrogen receptors and Ber-EP4 were the markers with the best performance in differentiating epithelioid peritoneal mesothelioma from serous papillary carcinoma of the ovary. Nasit et al. [128] reported that calretinin, WT-1, D2-40, h-caldesmon and thrombomodulin were the best positive markers for mesothelioma. More recently, the loss of immunohistochemical expression of BAP1 has been suggested to strongly support the diagnosis of mesothelioma in women presenting with abdominal disease [113,125,126]. Ito et al. [130] proposed the detection of the homozygous deletion of p16/CDKN2A (p16) by FISH as an effective tool for the diagnosis of malignant mesothelioma, which is now also considered a prognostic factor [30]. Moreover, new chromosomal mutations in mesothelioma have also been suggested as sensitive and specific diagnostic tools in mesothelioma [131].

### 4.3. Asbestos and Contaminated Talc vs. Primary Ovary Mesothelioma and Ovarian Cancer

The pathogenetic role of asbestos and contaminated talc in these forms is still debated. 

Asbestos exposure has been identified and debated as a possible risk factor for the development of ovarian cancer in some previous reviews and epidemiological studies [1,132,133,134,135,136,137,138,139,140,141,142,143,144,145,146,147].

The first evidence of this association dated back to 1967 when Graham et al. [10] showed for the first time the association between asbestos exposure and ovarian cancer followed by the results obtained by Newhouse et al. [133]. A high rate of death from ovarian cancer was found in women who worked in close contact with asbestos in several countries [143,144,148,149,150,151,152,153,154,155,156,157,158,159,160,161,162,163,164].

An increased risk of cancer incidence and mortality for ovarian cancer was also identified in women exposed to blue asbestos (crocidolite) during childhood at Wittenoom [165,166]. Henley et al. [167] examined the geographic co-occurrence of mesothelioma and ovarian cancer incidence rates in the US during 2003–2015. The authors reported a linear correlation between the two malignancies, indirectly providing the carcinogenic role of asbestos in both diseases. The hypothesis of a dose-dependent effect of asbestos in causing neoplastic degeneration was studied in former German asbestos workers, but the results were inconclusive [168].

The ultrastructural detection of a large amount of asbestos fibers (chrysotile and crocidolite) in ovaries and fallopian tubes was demonstrated by Heller et al. [53,169] and Langseth et al. [36]. Interestingly, most women were exposed to asbestos through household contacts with asbestos workers, by sexual relations (transvaginal route during coitus) [170] and by cosmetic perineal talc contaminated with asbestos [13].

The association between asbestos exposure, independent of the type [170], and the development of lung, laryngeal and ovarian cancers was finally confirmed by the Monograph 100c of the International Agency for Research on Cancer (IARC) [171].

Asbestos fibers persist in ovarian tissue [36,53], and they may likely reach the ovaries through retrograde movement in the reproductive tract [53,172]. Alternatively, the fibers may cross the mesothelium or the bladder wall and penetrate the ovary. In women, the peritoneum is ‘open’ at the ostium of the fallopian tubes, thus providing easily accessible entry of asbestos into the pelvic peritoneum and on the ovarian surface [16]. The persistence of fibers leads to chronic inflammation with macrophage activation, tissue injury, generation of oxygen reactive species, generation of nitrogen reactive species, genotoxicity, aneuploidy, polyploidy, epigenetic alteration, activation of signaling pathways and avoidance of apoptosis [173].

Concerning the role of contaminated talc, the first reports that suggested cosmetic talc use in the genital region as a possible factor for ovarian cancer date back to the 1970s [12,174,175], and until 1976 cosmetic talc was frequently contaminated by asbestos [31,47,48,49,50,51]. However, this remains an open debate with not yet conclusive results. [176,177,178,179,180,181].

Despite the FDA in 1973 had proposed using a polarizing microscope to ascertain a purity of talc at least 99.9%, manufacturing companies opted for the label “asbestos not detected” instead of “asbestos free”, considering this methodology not feasible [50]. In addition, the FDA found asbestos in cosmetic talc until 2018 when on 52 samples analyzed asbestos fibers were found to be present in 9 samples. The products were selected based on various factors including type of talc-containing powder cosmetic products, price range (i.e., low-end to high-end products), popular products on social media and in advertisements, children’s products, as well as certain products that had been reported by third parties to be contaminated with asbestos. On May 2020 a well-known manufacturer of talc-based baby powder withdrew the product from the sale in the United States. [182]. Apart from the contamination of talc with asbestos until the end of the 1970s and beyond [183,184], the presence of tainted talc in commercially available powder formulations has varied over time in various brands of products, resulting in difficulties for epidemiological studies to define an ascertained genital talc exposure [185] and to avoid statistical bias [186].

Several epidemiologic studies have evaluated the correlation between the use of perineal talc and the development of ovarian cancer [13,187,188,189,190,191,192,193,194,195,196,197,198,199,200,201,202,203]. A significantly higher risk of ovarian cancer was found in several studies [13,192,204,205,206,207]. In 2006, the International Agency for Research Cancer declared talc a possible carcinogenic factor for humans (group 2B) [172,208,209,210]. In 2008, Langseth et al. [211] reviewed 21 studies in which the perineal use of talc was found to be associated with ovarian cancer risk and suggested that the mechanism of carcinogenicity could be related to inflammation due to the latency between the age of the first use and the dose of exposure. Interestingly, a significantly increased risk was also found in women affected by borderline tumors of the ovary [212].

The debate on the role of talc as a carcinogen has continued until now. In 2011, Huncharek and Muscat [213] applied Bradford Hill’s criteria to epidemiological studies and concluded that a causal association between perineal talc use and ovarian cancer was not sufficiently proven. In contrast, Terry et al. [185] analyzed 8525 cases and 9859 controls, and they found that genital powder use was associated with a small-to-moderate increase in the risk of epithelial ovarian cancer. Gordon et al. [214] further investigated talc by electron microscopy. The type and number of fibers that could potentially be inhaled during normal use of talcum powder were found to be superimposable on those detected in the lung tissue of a woman who died of mesothelioma. The debate sparked by this paper led to several works focusing on this topic until last year with controversial results [35,37,184,215,216,217,218,219,220,221,222,223,224]. For example, it should be emphasized that no excesses of pleural mesothelioma nor talc pneumoconiosis were found in studies of miners in talc mines [177,225,226,227,228]. 

Similar to what was aforementioned for asbestos, talc applied to the genital area or on sanitary napkins, diaphragms or condoms may reach the ovary through retrograde movement in the reproductive tract [229,230], having been detected in benign and malignant ovarian tissue [36,191,231]. Alternatively, during ovulation, entrapment of the surface epithelium of the ovary into the ovarian stroma occurs, and either talc or other particulates might be incorporated into these inclusion cysts, acting as an “incessant ovulation” [232,233]. Moreover, some occupational studies have also suggested the migration of inhaled talc particles from the lung to the ovary [234]. The mechanism of carcinogenicity may be related to chronic inflammation that develops due to the persistence of talc crystals [235,236]. A certain role of estrogen and/or prolactin on macrophages and the inflammatory response to talc was also suggested [219,237].

The present study has several limitations. The unavailability of sufficient material and the low number of cases did not allow comprehensive clinic-pathological correlations or advanced molecular analyses. However, the clinical course, the whole histological, immunohistochemical, and ultrastructural features led us to be more in favor of a mesothelial origin rather than other neoplasms. The observation in two cases of crystals and inorganic material probably attributable to talc and tremolite does not claim to demonstrate a certain causal link between exposure and development of ovarian mesothelioma but could represent a starting point for more in-depth future mechanistic and epidemiologic studies that may or may not confirm the role of exposure in these rare neoplasms. Furthermore, the reported cases underline the importance of the mesothelioma registry both from an epidemiological point of view and in promoting research projects for the evaluation of the association between cases of mesothelioma and asbestos exposure.

## 5. Conclusions

In our case series, we described four cases diagnosed with primary ovarian mesothelioma.

The rarity of this entity and the difficulties to be differentiated either from ovarian carcinoma or peritoneal mesothelioma may lead to frequent misdiagnoses with underestimation of this tumor and may raise some concerns about their histogenesis.

Ovarian tumors and mesothelioma are strictly embryologically related, and both can be associated with asbestos or contaminated talc exposure. Nevertheless, the injury caused by the persistence of asbestos fibers and talc crystals may lead to the same carcinogenetic pathway occurring in mesotheliomas of other sites, thus reinforcing the existence of this kind of mesothelioma.

A combination of histological findings, immunohistochemical staining and electron microscopy analyses is mandatory to distinguish the different entities as relevant for correct patient care, for the choice of treatment and for prognosis. Thus, the report of such rare cases is fundamental to gain greater awareness of this neoplasm, to try and answer unsolved questions and to stress the importance of taking a comprehensive exposure history in these patients.

## Figures and Tables

**Table 1:** Main clinical features and exposure history of the four patients affected by ovarian mesothelioma.

Case Number	Year of Diagnosis	Year of Birth	Age at Diagnosis (Year)	Year of Death	Survival (Months)	Exposure (Calendar Years)	Duration of Exposure (Years)	Latency (Years)
1	1995	1957	38	lost to follow up (2005)	120 *	domestic (1975–1985)	10	20
2	1998	1933	65	2003	48	domestic (1938–1980 cosmetic talc)	42	60
3	2001	1947	54	2001	6	domestic (1947–1985 cosmetic talc)	38	54
4	2007	1955	52	lost to follow up (2010)	36 *	Familial (1981–1990)	9	26

* lost to follow up.

**Table 2:** Morphological, immunohistochemical and ultrastructural features of ovarian mesothelioma.

Case Number	Year of Diagnosis	Histological Diagnosis (TNM)	IHC	IHC/FISH Review 2020	TEM Ovarian Tissue Analysis	SEM Ovarian Tissue Analysis	Pathological Progression
1	1995	Bilateral well-differentiated papillary primary ovarian mesothelioma (T1b)	CA125−, EMA+++ vimentin+− −, calretinin+++	CDKN2a− BAP1− CEA−, MOC 31− Calretinin+++ WT1+++ D2-40 +++	Surface neoplastic cell with microvilli-like expansions and conspicuous cytoplasmic irregularity.	Talc crystals, fragments and an artificial fiber of glass wool, fragments of quartz. No asbestos fibers were identified.	Not available
2	1998	Bilateral biphasic malignant ovarian mesothelioma predominantly well differentiated papillary epitheliomorphic without invasive aspects(T1b)	Cytokeratin CK22 KL1+++ HMBE1+++ Vimentin+−−	CDKN2a– BAP1− CEA− MOC 31− Calretinin+++ WT1+++ D2-40+++	Neoplastic cells delimiting a fissure superficial with numerous microvilli. Cell of mesothelial type with microvilli and deep cytoplasmic incisions. Presence of intermediate filaments (cytokeratin) in place paranuclear.	Many crystals of talc, amphibole fiber tremolite, artificial fragments and fibers of glass wool, quartz crystals.	Pleural plaques (2000), secondary pleural and peritoneal mesothelioma (2002)
3	2001	Primary epithelial mesothelioma of the left ovary with papillary aspects of the ovarian cortex(T1b)	Cytokeratin+++ Vimentin+−− Calretinin+++ CD15− CEA−	BAP1− CEA− MOC 31− Calretinin+++ WT1+++ D2-40+++	Not available not executable	Not available not executable	Secondary malignant tumor of the peritoneum diffuse miliary peritoneal carcinosis, 12 months after the diagnosis
4	2007	Malignant epithelial mesothelioma with deciduous aspects involving the surface of the right ovary(T1b)	Desmin− Calretinin+++ WT1 +++ AE1-AE3 +++	BAP1− CEA− MOC 31− Calretinin+++ WT1+++ D2-40+++	Not available not executable	Not available not executable	Not available

**Table 3:** List of the twenty-one “ovarian mesothelioma” cases reported in the literature.

Year	Author	Cases Number	Age (Years)	Ovary Site	Asbestos Exposure	Reference Number
1972	Ferenczy	2	44	Right	Not Investigated	[64]
1979	Russell	2	42	Right	Not Investigated	[65]
			74	Left	Not Investigated	
1983	Addis and Fox	1	67	Left	No	[66]
1988	Hirakawa	1	61	Left	Not Investigated	[67]
1989	Vazquez	1	41	Bilateral	Not Investigated	[68]
1995	Goldblum	5	38	Bilateral	No	[69]
			58	Bilateral	No	
			71	Left	No	
			47	Bilateral	No	
			72	Bilateral	No	
1996	Clement	2	16	Right	No	[25]
			61	Bilateral	No	
2000	Attanoos and Gibbs	4	47	Right	No	[16]
			61	Left	Yes	
			66	Left	No	
			66	Right	Yes	
2006	Oh	1	43	Right	No	[70]
2017	Wills	1	41	Right	No	[71]
2019	Sun	1	50	Left	No	[72]

## Data Availability

Data sharing is not applicable to this article.
